# The effect of cholesterol overload on mouse kidney and kidney-derived cells

**DOI:** 10.1080/0886022X.2017.1419974

**Published:** 2018-01-05

**Authors:** Shoko Honzumi, Miho Takeuchi, Mizuki Kurihara, Masachika Fujiyoshi, Masashi Uchida, Kenta Watanabe, Takaaki Suzuki, Itsuko Ishii

**Affiliations:** a Department of Clinical Pharmacy, Graduate School of Pharmaceutical Sciences, Chiba University, Chiba, Japan;; b Division of Pharmacy, Chiba University Hospital, Chiba, Japan

**Keywords:** Beta-very low density lipoprotein, megalin, renal proximal tubule epithelial cell, renal lipotoxicity, dyslipidemia

## Abstract

**Introduction:** Dyslipidemia is one of the onset and risk factors of chronic kidney disease and renal function drop is seen in lipoprotein abnormal animal models. However, the detailed molecular mechanism of renal lipotoxicity has not been clarified. Therefore, the present study aimed to investigate the influence of cholesterol overload using mouse kidney tissue and kidney-derived cultured cells.

**Methods:** C57BL/6 mice were fed normal diet (ND) or 1.25% cholesterol-containing high-cholesterol diet (HCD) for 11 weeks, and we used megalin as a proximal tubule marker for immunohistology. We added beta-very low density lipoprotein (βVLDL) to kidney-derived cells and examined the effect of cholesterol overload on megalin protein and mRNA expression level, cell proliferation and cholesterol content in cells.

**Results:** In the kidney of HCD mice, the gap between glomerulus and the surrounding Bowman’s capsule decreased and the expression level of megalin decreased. After βVLDL treatment to the cells, the protein expression and mRNA expression level of megalin decreased and cell proliferation was restrained. We also observed an increase in cholesterol accumulation in the cell and free cholesterol/phospholipid ratios increased.

**Conclusions:** These findings suggest that the increased cholesterol load on kidney contribute to the decrease of megalin and the overloaded cholesterol is taken into the renal tubule epithelial cells, causing suppression on cell proliferation, which may be the cause of kidney damage.

## Introduction

Dyslipidemia is one of the onset and risk factors of not only cardiovascular disease, but also of the progression of renal failure in human chronic renal disease [1,2]. It is known that lipoprotein abnormalities aggravate renal injury via podocyte, a glomerulus epithelial cell, and that such podocyte injury is accompanied by tubulointerstitial cell activation and cell injury in animal models [3]. It has also been shown that elevated levels of lipids accelerate renal disease progression, which can be improved by a variety of manipulations that lower circulating lipids or prevent intracellular lipid accumulation [4–7]. High-triglycerides, high non-high density lipoprotein (non-HDL) cholesterol, low-HDL cholesterol, elevated total cholesterol (TC) and high-apolipoprotein B (ApoB) are reported to influence the decline in renal function [8–11].

The mechanism by which lipids affect kidneys support the lipid nephrotoxicity hypothesis proposed by Moorhead et al. in 1982 [12], is widely supported. According to this hypothesis, circulating low-density lipoprotein (LDL) binds with glycosaminoglycan in the glomerulus basement membrane, and increases its permeability. Lipid abnormalities contribute to both atherosclerosis and glomerulosclerosis as cholesterol relocates to and accumulates in renal, vascular, hepatic and possibly other tissues [13]. Moorhead et al. [12] reported that filtered lipoprotein accumulates in mesangial cells. Diamond et al. [14] proposed that the infiltration of the macrophage into the mesangium participates in the proliferation of mesangial cells, and the glomerulus capillary endothelial damage by the lipoprotein deteriorates the hemodynamics in the kidney, leading to renal damage progression.

Megalin, or LDL receptor-related protein 2 (LRP2), is a member of the LDL receptor (LDLR) family and is an endocytic receptor expressed on the apical surface of several epithelial cells that internalizes a variety of ligands [15]. Megalin is considered to define the apical recycling pathway of epithelial cells and to have an influence on the high-protein absorption capacity of the kidney proximal tubule, and on genetic proteinuric syndromes [16]. Megalin is involved in the uptake of drugs and toxic substances such as denatured protein, and also in the renal tubule cell disorder by the excessive load of plasma protein [17–19].

Most examinations about renal damage due to hyperlipidemia are conducted using animal disease models [20], and there are only a few high-cholesterol diet (HCD) fed models. Tomizawa et al. [21] have reported a renal function change by magnetic resonance imaging (MRI) analysis in the kidneys of HCD-induced kidney dysfunction mice [22,23]. HCD mice showed significantly higher levels of blood urea nitrogen, creatinine and uric acid compared to the normal diet (ND) mice [24]. Using carbamoyl-PROXYL (CMP) as the contrast probe, the MRI signal increased after the injection of CMP in the ND mice, although in HCD mice, CMP-mediated enhancement of the MRI signal was not detected [24,25]. However, when the mice were treated with antilipidemic drugs, which lowers cholesterol levels in the blood, the MRI signal appeared very similar to that in the ND mice. Therefore, a possibility exists that the increase in the blood cholesterol affects the renal dysfunction.

It is considered that dyslipidemia causes renal damage, however, the detailed molecular mechanism has not been clarified. In the present study, we examined the influence of cholesterol load on kidney by using mouse kidney tissue and kidney-derived cultured cells.

## Materials and methods

### Histological examination of mouse kidney

Male C57BL/6 mice were fed ND or HCD (1.25% (*w/w*) cholesterol-containing high-cholesterol diet, Oriental Yeast Co., Tokyo, Japan) for 11 weeks. After 11 weeks, their kidneys were extracted and made into paraffin-embedded sections and they were stained by Masson’s trichrome staining. We used megalin as a proximal tubule marker and examined the renal tubular change for immunohistology. Immunostaining was conducted using a VECTOR (VECTASTAIN Elite ABC Kit, Burlingame, CA, USA) with megalin polyclonal antibody (P-20, Santa Cruz Biotechnology, Santa Cruz, CA, USA) according to the manufacturer’s protocol.

### Western blot of megalin in mouse kidney homogenate and cultured cells

Male C57BL/6 mice were fed ND or HCD for 12 weeks. Kidneys were extracted after perfusion, IP buffer A (20 mM Tris-HCl [pH 7.4], 150 mM NaCl, 0.5% Triton X-100, 0.5% sodium deoxycholate, 10 mM EDTA, 0.2% protease inhibitor cocktail) was added and homogenized. After centrifugation, the supernatant was collected and used for Western blot analysis. For cultured cells, cells were collected by trypsin-EDTA solution treatment and dissolved in IP buffer B (20 mM Tris-HCl [pH 7.4], 150 mM NaCl, 0.5% Triton X-100, 0.5% sodium deoxycholate, 10 mM EDTA, 0.1 mM FUT-175, 0.1 mM E-64-C, 0.5 mM Na_3_VO_4_, 5 mM NaF) and the supernatant was collected by centrifugation for Western blot analysis. Western blot analysis was carried out using megalin polyclonal antibody as described above according to the manufacturer’s protocol.

### Effects of beta-migrating very low-density lipoprotein (βVLDL) on megalin in kidney-derived cultured cells

βVLDL was prepared by ultracentrifugation according to the method by Goldstein, et al. [1,26] from blood serum of New Zealand white rabbits (Takasugi Experimental Animals Supply Co., Ltd., Kasukabe-shi, Japan) fed RC-4 containing 1% cholesterol (Oriental Yeast Co., Tokyo, Japan).

For kidney-derived cultured cells, we used epithelial cell lines originally derived from porcine kidneys (LLC-PK1 [DS Pharma Biomedical, Suita-shi, Japan]), a mouse renal mesangial cell line (SV40 MES 13 [ATCC]), a human renal mesangial cell (HRMC [ScienCell Research Laboratories, Carlsbad, CA, USA]) and a human renal proximal tubular epithelial cell (HRPTEC [ScienCell Research Laboratories]). They were maintained according to the manufacturer’s protocol and βVLDL were added at 0.2 mg TC/mL on the day after being plated.

For RNA analysis, total RNA was extracted using an QIAGEN (RNeasy Mini Kit, Hilden, Germany) and cDNA syntheses were performed using a high-capacity RNA-to-cDNA Kit (Applied Biosystems, Foster City, CA, USA). A real-time quantitative PCR (RT-PCR) assay was performed using an Applied Biosystems 7900 sequence detector with TaqMan Gene expression assays (Applied Biosystems). The assay IDs were as follows: human peptidylprolyl isomerase B (PPIB), Hs00168719_m1; human megalin, Hs00189742_m1. Relative mRNA levels were normalized to PPIB mRNA levels.

### Effect of βVLDL on cellular proliferation and cholesterol accumulation

LLC-PK1 and MES 13 were incubated with or without βVLDL. The cells were dyed with Trypan blue stain 0.4% (Life Technologies Corporation, Carlsbad, CA, USA) according to the manufacturer’s protocol and the cell number was counted to evaluate cellular proliferation.

For cholesterol accumulation, LLC-PK1 and MES 13 were treated with βVLDL for two days. After the βVLDL treatment, cells were collected and lipids were extracted using hexane: isopropanol (2:1, *v/v*). TC was measured using a Cholesterol *E*-test (Wako Pure Chemical Industries, Ltd, Osaka-shi, Japan), free cholesterol (FC) was measured using a free Cholesterol *E*-test (Wako Pure Chemical Industries, Ltd) and cholesterol ester (CE) was calculated by subtracting FC from TC.

### Dose-dependent accumulation of lipid and localization of neutral lipid

LLC-PK1 and MES 13 were treated with 0, 0.2, 0.5 and 1 mg TC/mL βVLDL for two days. Lipids were extracted as described above and phospholipid (PL) was measured using a phospholipid *C*-test (Wako Pure Chemical Industries, Ltd).

The intracellular distribution of neutral lipid and FC after two days of treatment with or without βVLDL was examined using Oil Red O staining and filipin staining, respectively according to the manufacturer’s protocol.

### Statistical analysis

The results are expressed as the mean ± standard error (SE). Statistical analyses were conducted with SAS System Release 8.2 (SAS Institute Inc., San Francisco, CA, USA). Comparisons between ND and HCD groups were performed with Student’s *t*-test. A value of *p* < .05 was considered significant.

## Results

### Histological examination of mouse kidney

**Figure 1.**
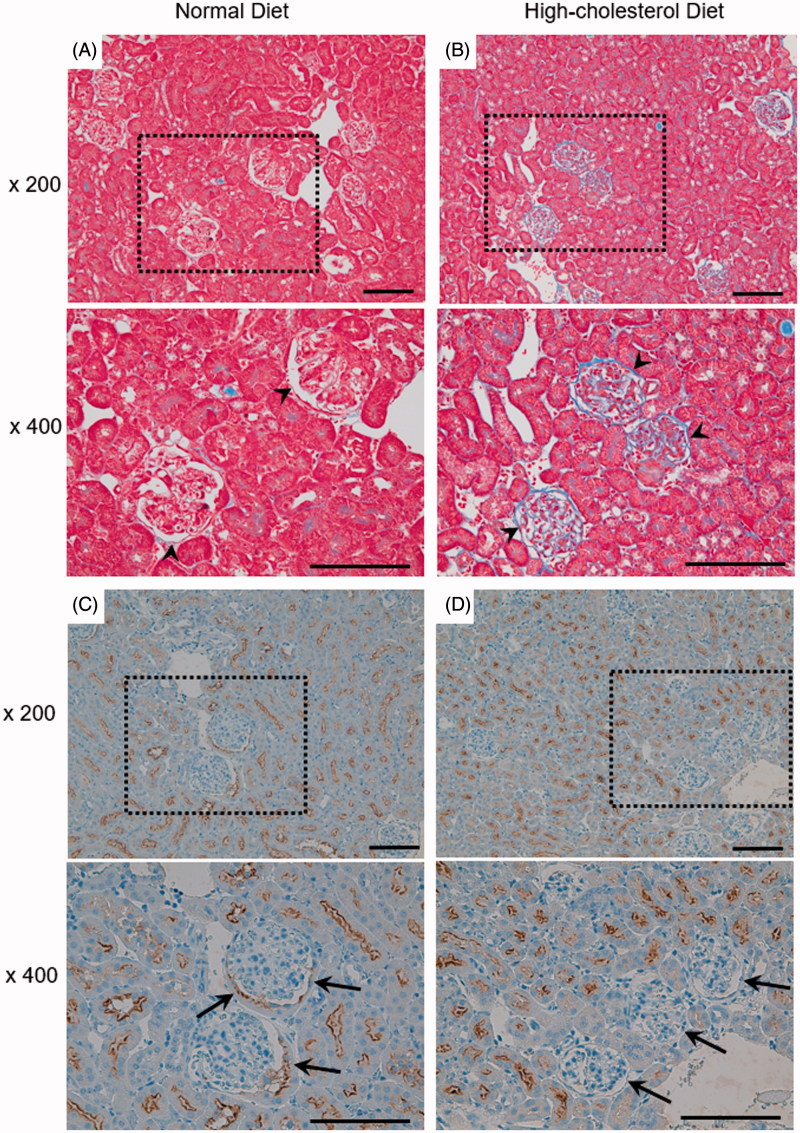
Histological examination of mouse kidney. Male C57BL/6 mice were fed a normal diet (ND; A,C) or high-cholesterol diet (HCD; B,D) for 11 weeks. (A,B) Representative micrographs showing Masson’s trichrome staining. Arrowhead: Bowman’s space. (C,D) Representative micrographs showing immunostaining with megalin (proximal tubule marker, dark staining). Arrow: tubular epithelium lining of the proximal tubules which are contiguous with Bowman’s capsule. Box area is enlarged to compare HCD with ND (lower panels). Scale bars: 50 μm.

Representative micrographs show Masson’s trichrome staining of the kidney (Figure 1(A,B)). In the HCD mice, the gap between the glomerulus and the surrounding Bowman’s capsule decreased. To examine the structural change of the renal tubule, we performed immunohistochemistry dyeing of megalin (Figure 1(C,D)). The expression of megalin in tubular epithelium lining the proximal tubules that are contiguous with Bowman’s capsule decreased in the HCD mice in comparison with the ND mice.

### Western blot of megalin in mouse kidney homogenate

**Figure 2.**
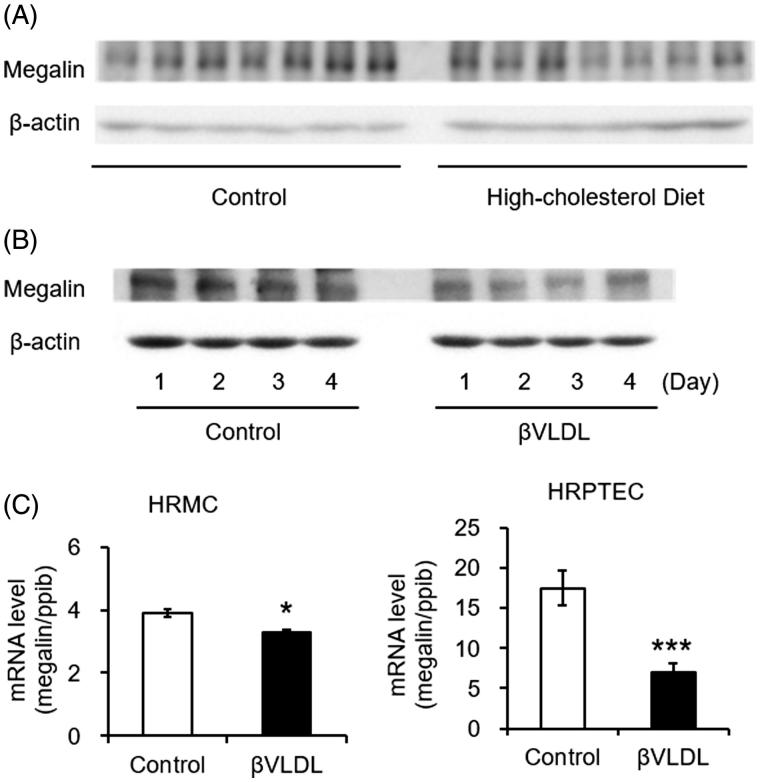
Western blot of megalin in mouse kidney homogenate and effects of βVLDL on megalin in kidney derived cultured cells. (A) Male C57BL/6 mice (*n* = 7 per group) were fed normal diet or high-cholesterol diet for 12 weeks. Kidney extracts were used to determine the protein levels of megalin by Western blot. (B) LLC-PK1 was incubated for the indicated number of days with or without 0.2 mg TC/mL βVLDL. (C) HRMC and HRPTEC were incubated with or without 0.2 mg TC/mL βVLDL for two days in triplicate. Each bar represents the mean ± SE, *p < .01, ***p < .001, as compared with control.

To examine the influence of HCD on the expression of megalin in the kidney tissue, kidney extracts were used to determine the protein levels of megalin by Western blot analysis (Figure 2(A)). When the band of each group in the blots was quantified by densitometry, megalin expression was lower in HCD fed mice than in the ND mice, but there was no statistically significant difference between the two groups.

### Effects of βVLDL on protein levels and mRNA levels of megalin in kidney-derived cultured cells

To consider whether cholesterol load influences the megalin expression in the proximal tubule epithelial cells, the expression of megalin in βVLDL-loaded LLC-PK1 cells was detected by Western blot analysis (Figure 2(B)). As a result, there was less megalin expression in the βVLDL-loaded cells compared to cells without treatment. To investigate the influence of cholesterol load, HRPTEC and HRMC were treated with βVLDL for two days and the mRNA levels were detected by RT-PCR (Figure 2(C)). The mRNA levels of megalin were significantly reduced by the treatment with βVLDL.

### Effect of βVLDL on cellular proliferation and cholesterol accumulation

**Figure 3.**
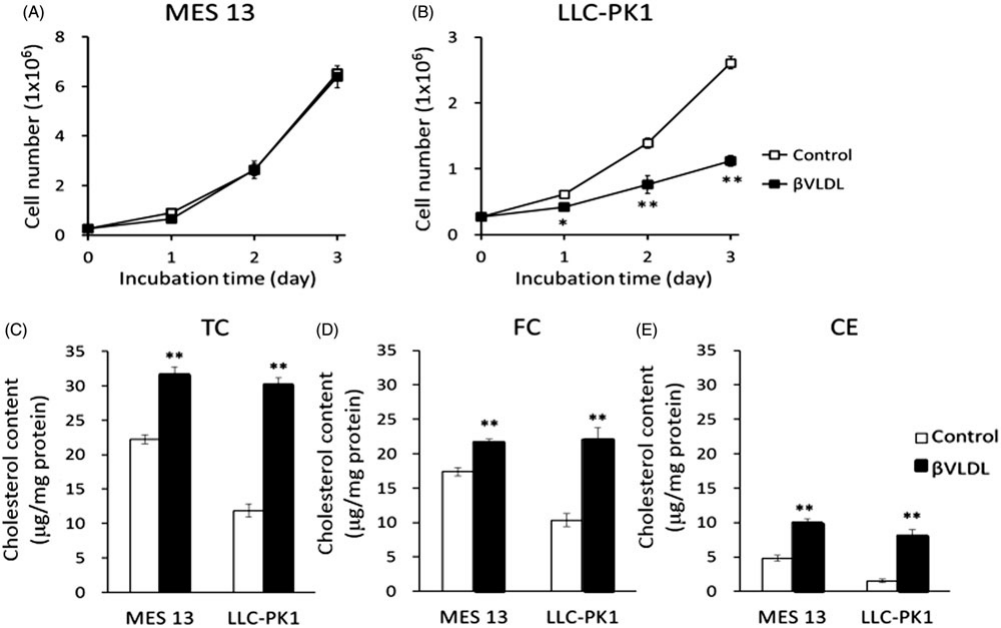
Effect of βVLDL on cellular proliferation and cholesterol accumulation. Cells were incubated with or without 0.2 mg TC/mL βVLDL for the indicated number of days. The cell number was counted. (A) MES 13; (B) LLC-PK1. Each point represents the mean ± SE. (C,D) Cells were incubated for two days with or without 0.2 mg TC/mL βVLDL. Intracellular TC and FC were determined by enzymatic colorimetric assays. The concentration of CE was determined by subtracting FC from TC (E). Each bar represents the mean ± SE from triplicates. *p < .05, **p < .01, as compared with control.

We examined the influence of βVLDL on the cellular proliferation of MES 13 (Figure 3(A)) and LLC-PK1 (Figure 3(B)). By counting the cell numbers, the cell number of MES 13 did not change by adding βVLDL, but the cell number of LLC-PK1 was significantly decreased.

To investigate the mechanism of growth suppression by βVLDL in LLC-PK1, we checked the cholesterol accumulation on day 2, when the cell number difference was great. In MES 13, TC, FC and CE significantly increased to approximately 1.4-fold, 1.2-fold and 2.1-fold, respectively, by βVLDL load. In LLC-PK1, TC, FC and CE significantly increased to approximately 2.5-fold, 2.1-fold, and 5.3-fold, respectively (Figures 3(C–E)).

### Dose-dependent accumulation of lipid and localization of neutral lipid

To consider the component change of the cell membrane of LLC-PK1, in which the restraint of the cell proliferation was seen by βVLDL load, we checked the changes in the amounts of PL, the main component of the cell membrane. In MES 13, the quantity of FC and PL was approximately constant regardless of the concentration of the loaded βVLDL (Table 1). On the other hand, in LLC-PK1, FC and PL increased depending on the concentration of the loaded βVLDL. In addition, after calculating the molar ratio of FC to PL (FC/PL), the change of the value by the loaded βVLDL concentration was greater in LLC-PK1 than in MES 13.

**Table 1.** Dose-dependent accumulation of lipid in MES 13 and LLC-PK1 cells.

	βVLDL	FC	PL	FC/PL
	(mg TC/mL)	(μmol/mg protein)	(μmol/mg protein)	Molar ratio
MES 13	0.0	0.05 ± 0.00	0.09 ± 0.00	0.57
	0.2	0.06 ± 0.00	0.08 ± 0.00	0.70
	0.5	0.06 ± 0.01	0.08 ± 0.09	0.70
	1.0	0.06 ± 0.00	0.08 ± 0.00	0.79
LLC-PK1	0.0	0.04 ± 0.00	0.08 ± 0.00	0.51
	0.2	0.06 ± 0.01	0.09 ± 0.01	0.68
	0.5	0.12 ± 0.03	0.11 ± 0.03	1.04
	1.0	0.11 ± 0.04	0.13 ± 0.05	0.86

Cells were incubated with βVLDL for two days at the indicated dosage. Each value represents the mean ± SE from triplicates. An average PL molecular weight of 800 was used in determining PL-to-protein weight ratios.

**Figure 4.**
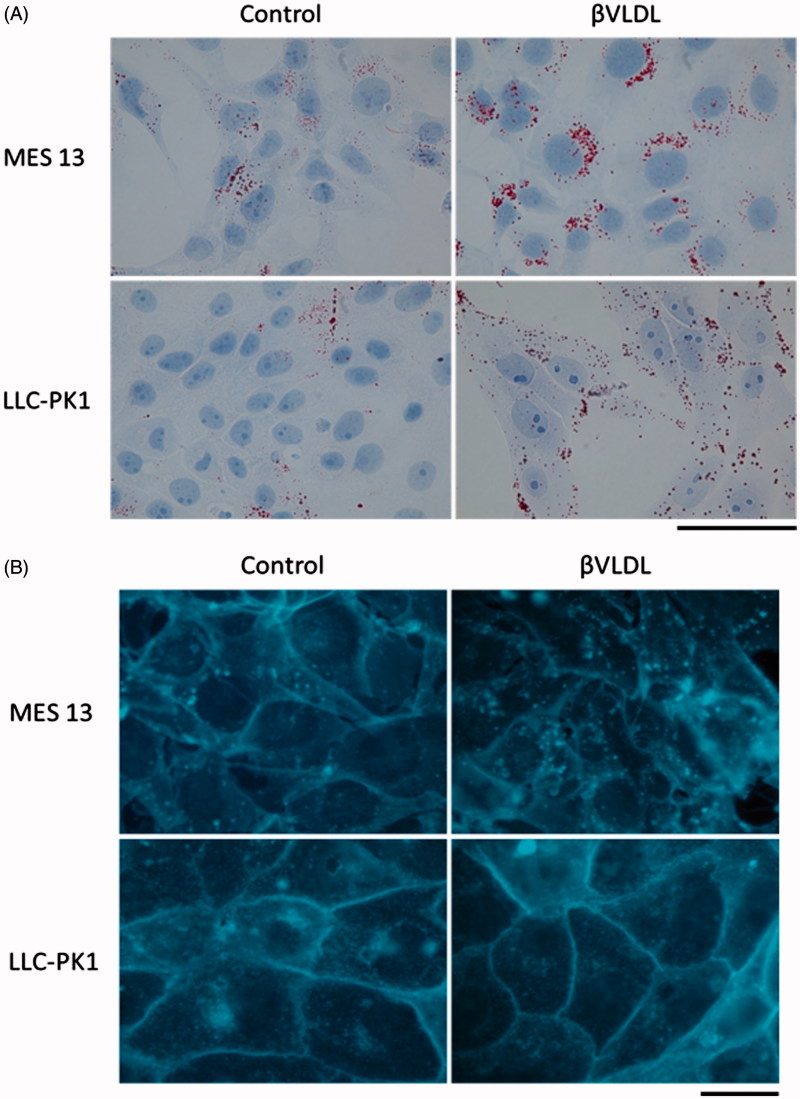
Localization of neutral lipid and FC in MES 13 and LLC-PK1 cells loaded with βVLDL. (A) The intracellular distribution of neutral lipid was examined using Oil Red O staining. (B) The intracellular distribution of FC was examined using filipin staining. Cells were incubated for two days with or without 0.2 mg TC/mL βVLDL. Bar; 20 μm.

To investigate the localization of neutral lipids and FC in the cells, we dyed the lipids with Oil Red O (Figure 4(A)) and FC with filipin (Figure 4(B)). In MES 13, more lipid accumulated near the nucleus, while in LLC-PK1 the lipid scattered within the cell. In MES 13, the FC was seen not only in the cell membrane but also in the cytoplasm, while in LLC-PK1 the FC was located more in the cell membrane. In both cells, cholesterol accumulation inside the cell was observed by βVLDL treatment.

## Discussion

In the kidney of the HCD mice, the gap between glomerulus and the surrounding Bowman’s capsule decreased (Figures 1(A,B)). Tomizawa et al. [24] have reported that HCD mice showed significantly higher levels of blood urea nitrogen, creatinine and uric acid compared to the ND mice. In HCD mice, Tomizawa et al. showed that the quantity of glomerulus filtration decreased. It is considered that the decrease in quantity of glomerulus filtration causes the filtration pressure of the remaining individual nephrons to rise [27], which may have affected the size of the gap between the glomerulus and the surrounding Bowman’s capsule. It is reported that the damage of podocyte causes leakage of protein from the basement membrane, inducing further damage to the podocyte and causing loss of podocyte, leaving bare basement membrane. Parietal epithelial cells attach to the bare basement membrane, leading to the formation of a tuft adhesion to the Bowman’s capsule [28,29]. LeHir and Kriz [30] proposed that cellular adhesion was formed predominantly by injured podocytes. From the above, there is a possibility that the decreased gap between the glomerulus and the Bowman’s capsule by cholesterol overload enhances the adhesion to Bowman’s capsule.

In our study, the expression level of megalin in the Bowman’s capsule junction of the proximal tubule of HCD mice decreased (Figures 1(C,D)). Bowman’s capsule junction is the first part through which the primitive urine, after already having passed the glomerulus, flows into the renal tubule, and it is also the part that is most easily affected by the protein in the primitive urine. Proteinuria is observed in megalin-deficient mice, therefore megalin is thought to take part in proximal tubule protein reabsorption of the glomerulus-filtrated protein [18,31]. The decrease of megalin observed in the Bowman’s capsule junction may have an effect on proximal tubule protein reabsorption, leading to renal function deterioration.

Serum apoB-100, apoB-48 and apoE expression level was higher in HCD mice compared to ND mice (data not shown). Since βVLDL is loaded with apoB-100, apoB-48 and apoE [32,33], we used βVLDL for treating the cells to evaluate the influence of cholesterol overload. When we treated LLC-PK1 with βVLDL, the expression level of megalin was lower compared to that of non-treated controls (Figure 2(B)). The mRNA levels of megalin in HRPTEC and HRMC was also decreased by βVLDL load (Figure 2(C)). It is reported that accumulation of cholesterol in the mesangial region causes glomerular sclerosis due to foam cell formation of mesangial cells and expansion of glomerulus basement membrane [34]. We found a decrease in megalin expression in HRMC and our finding suggests a possibility that the decrease in megalin affect the expansion of glomerulus basement membrane, leading to glomerular sclerosis. Altogether, it is considered that apoB and apoE, which are increased in blood by HCD feeding, leak into the primitive urine and may be the reason for the decrease of megalin expression in the Bowman’s capsule junction. Since apoB and apoE are also the ligands for megalin [19,35], the decrease of megalin by βVLDL load may also be one of the self-protective mechanisms of the renal tubule cells.

Cellular proliferation was restrained by βVLDL load in LLC-PK1 (Figure 3(B)). We considered the possibility that βVLDL induced apoptosis in LLC-PK1. We checked the activation of caspase-3 and the fragmentation of DNA as an index of the apoptosis. However, treatment by βVLDL did not induce apoptosis in LLC-PK1. We also checked the effect of βVLDL on cell cycle progression in HRPTEC by Western blot and RT-PCR of cyclin-dependent kinase (CDK) and cyclin, and the mRNA levels of CDK2, CDK4 and cyclin D3 were decreased (data not shown). These results suggest that the decrease in the cell cycle progression genes may be responsible for the proliferation suppression by βVLDL in LLC-PK1.

TC, FC and CE increased (Figures 3(C–E)) and cholesterol accumulated in cells by βVLDL load in MES13 and LLC-PK1 cells (Figures 4(A,B)). The molar ratio of FC to PL (FC/PL) changed from 0.57 in non-treated cells to 0.79 in βVLDL-loaded MES13, and from 0.51 to maximum of 1.04 in LLC-PK1 cells (Table 1). An increase in FC/PL decreases membrane fluidity [36,37], and cholesterol has been found to modulate the function of membrane proteins critical to cellular function [38]. Therefore, the accumulation of FC and the increase in the FC/PL may have also affected the cell proliferation of LLC-PK1.

In summary, we examined the effect of cholesterol load on kidney by using mouse kidney tissue and kidney-derived cultured cells. We detected a reduction in the gap between the glomerulus and the Bowman’s capsule, and a decrease in megalin expression in the proximal tubule of HCD mouse kidneys, suggesting that the increase of lipoprotein in the primitive urine may decrease the megalin expression in the proximal tubule epithelial cells. When we tested a βVLDL load against LLC-PK1, the cell proliferation of LLC-PK1 decreased. Since FC/PL increase was seen in LLC-PK1, there is a possibility that βVLDL load decreases the membrane fluidity, leading to the suppression of cell proliferation in tubular epithelial cells. Altogether, cholesterol overload may influence chronic kidney disease and decrease renal function by increasing the amount of cholesterol taken up into the proximal tubular epithelial cells, decreasing megalin and causing suppression on cell proliferation, which may be the cause of kidney damage.
